# Molecular evolution and phylogenetics of rodent malaria parasites

**DOI:** 10.1186/1471-2148-12-219

**Published:** 2012-11-14

**Authors:** Ricardo S Ramiro, Sarah E Reece, Darren J Obbard

**Affiliations:** 1 Institute of Evolutionary Biology, University of Edinburgh, Edinburgh, United Kingdom; 2 Institute of Immunology and Infection Research, University of Edinburgh, Edinburgh, United Kingdom; 3 Centre for Immunity, Infection and Evolution, University of Edinburgh, Edinburgh, United Kingdom

**Keywords:** Rodent malaria, *Plasmodium*, Phylogeny, Species delimitation, Divergence time, Molecular evolution

## Abstract

**Background:**

Over the last 6 decades, rodent *Plasmodium* species have become key model systems for understanding the basic biology of malaria parasites. Cell and molecular parasitology have made much progress in identifying genes underpinning interactions between malaria parasites, hosts, and vectors. However, little attention has been paid to the evolutionary genetics of parasites, which provides context for identifying potential therapeutic targets and for understanding the selective forces shaping parasites in natural populations. Additionally, understanding the relationships between species, subspecies, and strains, is necessary to maximize the utility of rodent malaria parasites as medically important infectious disease models, and for investigating the evolution of host-parasite interactions.

**Results:**

Here, we collected multi-locus sequence data from 58 rodent malaria genotypes distributed throughout 13 subspecies belonging to *P. berghei, P. chabaudi, P. vinckei,* and *P. yoelii.* We employ multi-locus methods to infer the subspecies phylogeny, and use population-genetic approaches to elucidate the selective patterns shaping the evolution of these organisms. Our results reveal a time-line for the evolution of rodent *Plasmodium* and suggest that all the subspecies are independently evolving lineages (i.e. species). We show that estimates of species-level polymorphism are inflated if subspecies are not explicitly recognized, and detect purifying selection at most loci.

**Conclusions:**

Our work resolves previous inconsistencies in the phylogeny of rodent malaria parasites, provides estimates of important parameters that relate to the parasite’s natural history and provides a much-needed evolutionary context for understanding diverse biological aspects from the cross-reactivity of immune responses to parasite mating patterns.

## Background

Since their discovery in Central West Africa in the 1940s, rodent malaria parasites - *Plasmodium berghei*, *P. chabaudi*, *P. vinckei* and *P. yoelii* – have become the *de facto* animal models for the study of *Plasmodium* biology [1]. These parasites have proven useful to investigate diverse aspects of host-parasite-vector interactions, evaluate potential interventions for malaria control, and to generate and test hypothesis about the biology of human malaria [1-3]. Although, no single species of rodent *Plasmodium* is the perfect model for human parasites, different species have proven useful for different aspects of biology [4]. For example, while *P. chabaudi* is a good model for the study of infection dynamics and immune responses [2], *P. berghei* is a better model for studying the biology of transmission [5,6]. Nevertheless, while the rodent malarias are well studied in the lab, much less is known about their natural history and evolutionary genetics. This is in sharp contrast to human pathogens, where evolutionary genetics plays an important role, particularly in explaining the evolution of drug resistance and patterns of natural selection at target antigens [7].

Rodent malaria parasites were isolated from the wild between 1948 and 1974, from 5 different countries (Cameroon, Central African Republic, Congo, Democratic Republic of the Congo and Nigeria) [1]. During this period, four species were described, encompassing 13 subspecies. The ‘thicket rats’ *Thamnomys rutilans* (also known as *Grammomys poensis*) and *Grammomys surdaster* (also known as *Grammomys dolichurus*) [1,8] were found to be the most common vertebrate hosts and whilst *Anopheles dureni millecampsi* was frequently infected by rodent malaria parasites, the vector for some of these parasite species remains unknown (reviewed in [1]; Additional file 1). Although only a handful of genotypes are regularly used in the lab, the World Health Organization (WHO) Registry of Standard Malaria Parasites (in the European Malaria Reagent Repository, University of Edinburgh) contains over 70 field isolates of rodent *Plasmodium*. The availability of multiple isolates means that multi-locus sequence data and modern phylogenetic methods can be used to improve understanding of many aspects of the natural history of rodent malaria parasites. This includes identifying lineages that are independently evolving (species delimitation), estimating the ‘species tree’ (phylogeny), divergence times, and population sizes [9]. Additionally, an improved phylogeny enables the better application of molecular evolution methods, as these often depend on an understanding of the species tree (e.g. [10]).

Despite the variety of papers on *Plasmodium* phylogeny, only one has addressed the phylogeny of rodent malaria parasites (e.g. [11,19,20]). Perkins *et al.*(2007) sequenced fragments of 7 loci from the nuclear, mitochondrial and apicoplast genomes for 19 parasite genotypes covering all the species and 12 of the subspecies of rodent *Plasmodium.* Their results were consistent with the original species descriptions based on morphological and isozyme characteristics, but revealed inconsistencies in the level of genetic divergence between genotypes, subspecies and species (e.g. pairwise divergence between genotypes of different *P. vinckei* subspecies was shown to be much higher than between genotypes of *P. berghei* and *P. yoelii*) [1,14]. Here, we collect the largest rodent malaria sequence dataset to date, to resolve these inconsistencies, improve the phylogeny and generate a resource for future population genetic and experimental studies in these important model species.

We revived 58 rodent *Plasmodium* genotypes (some for the first time in 44 years) from cryopreservation, representing 13 subspecies across four species and sequenced 11 ‘house-keeping’ nuclear loci. We use these data to understand species delimitation and generate a multi-locus phylogeny. We infer the relative effective population sizes, generate hypotheses for the divergence times and describe the patterns of selection and constraint in these loci. First, our results suggest that all rodent malaria subspecies are sufficiently genetically isolated that they could be considered species. Second, we estimate the (sub)species tree, divergence times and population sizes, under two different time-calibrations. The two alternative calibrations we used reflect current uncertainty in *Plasmodium* evolution and, as expected, resulted in very different divergence times. Finally, we estimate levels of polymorphism and calculate summary statistics that reflect the form and strength of natural selection (*K*_*A*_*/K*_*S*_, McDonald-Kreitman tests and Tajima’s D statistic). Our results show that polymorphism is low and reveal that most loci are under purifying selection.

## Results

We obtained 58 rodent malaria genotypes from the WHO Registry of Standard Malaria Parasites (European Malaria Reagent Repository, University of Edinburgh; Tables 1 and Additional file 1) and collected DNA sequence data for 11 nuclear loci (see methods), across 3 chromosomes, mainly coding for ‘house-keeping’ functions (GenBank accession no. in Table 2). Additionally, where explicitly mentioned, our analysis incorporated publically available data for cytochrome b (*cytb*) and cytochrome oxidase I (*coI*; GenBank accession no. *coI:* DQ414589–DQ414606; *cytb:* AY099050, AY099051, DQ414545– DQ414660; [14]).

**Table 1:** Rodent malaria species and subspecies selected for analysis

**species**	**subspecies**	**no. genotypes**	**country of origin**
*P. berghei*	-	8	DRC
*P. chabaudi*	*adami*	2	Congo
	chabaudi	14	CAR
	subsp.	2	Cameroon
*P. vinckei*	*brucechwatti*	2	Nigeria
	*lentum*	3	Congo
	*petteri*	3	CAR
	*vinckei*	2	DRC
	subsp.	5	Cameroon
*P. yoelii*	*killicki*	2	Congo
	*nigeriensis*	2	Nigeria
	*yoelii*	10	CAR
	subsp.	3	Cameroon

The number of genotypes we used from each species/subspecies and their country of origin is shown (see Additional file 1 for a more detailed description of the origin of each genotype). *P. c.* subsp., *P. v.* subsp. and *P. y.* subsp. refer to previously recognized, but as yet unnamed subspecies. CAR – Central African Republic, DRC – Democratic Republic of the Congo.

**Table 2:** House-keeping loci selected for analysis

**gene**	**chr**	**gene ID**	**predicted function**	**length (bp)**	**no. of sequenced genotypes**	**GenBank accession no.**
*26 s*	3	PBANKA_030360	proteasome 26S regulatory subunit	742	55	JX904678 - JX904732
*atpase*	13	PBANKA_136260	nucleolar preribosomal associated cytoplasmic ATPase	716	54	JX904733 - JX904786
*cons*	3	PBANKA_030790	conserved *Plasmodium* protein, unknown function	756	19	JX904787 - JX904805
*cyspro*	13	PBANKA_132170	cysteine proteinase	811	53	JX904806 - JX904858
*dhfr*	7	PBANKA_071930	bifunctional dihydrofolate reductase-thymidylate synthase	610	56	JX904859 - JX904914
*exonuc*	3	PBANKA_030260	3'-5' exonuclease	591	49	JX904915 - JX904963
*gdpgmp*	3	PBANKA_030880	GDP-fructose:GMP antiporter	256	50	JX984464 - JX984513
*glurna*	13	PBANKA_136200	glutamate - tRNA ligase	563	39	JX904964 - JX905002
*hsp70*	13	PBANKA_135720	heath shock protein 70	658	52	JX905003 - JX905054
*metrans*	3	PBANKA_030390	metabolite/drug transporter	685	43	JX905055 - JX905097
*rnabind*	13	PBANKA_135690	RNA-binding protein	554	56	JX905098 - JX905153

Gene ID and predicted function were obtained from PlasmoDB. Maximum sequence length (length); Chromosome (chr).

### Species delimitation

To better understand the status of the subspecies we used the following two methods for species delimitation: Bayesian species delimitation as described in [15] and genealogical sorting index, described in [16].

#### Bayesian species delimitation

We used the program Bayesian Phylogenetics and Phylogeography v2.0 (BPP, [15]) to perform species delimitation analysis. This implements a coalescent-based method that uses the concordance of gene trees across multiple loci as evidence for a particular species delimitation model, but does not rely on reciprocal monophyly for each individual locus. It uses a reversible-jump Markov chain Monte Carlo (rjMCMC) algorithm to calculate the posterior distributions of different species delimitation models. The method is based on the biological species concept, assuming no migration following speciation and allowing for stochastic fluctuations in the coalescent process and lineage sorting due to ancestral polymorphism [15,17,18].

**Figure 1:**
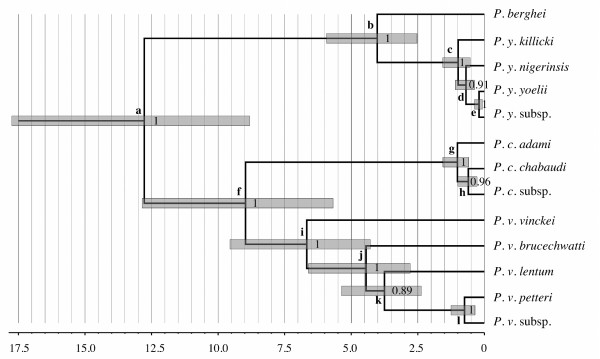
**Rodent malaria phylogeny and divergence times for the Pacheco2011-B calibration.** This *BEAST time-tree is an example of the divergence times we obtained, though the results from Pacheco2011-A should also be taken into account (see Table 3 and Additional file 9). Node bars represent 95% HPD and axis is in Mya. Internal node labels represent posterior support and external node labels correspond to the divergence times in Table 3. The topology of this tree was the same as the guide tree for BPP.

BPP requires a user-specified guide tree as input to constrain the phylogeny and species delimitation space. The guide tree is a fully resolved tree, representing the most subdivided species delimitation model that is biologically plausible. The rjMCMC algorithm then evaluates the posterior probabilities of speciation models created by collapsing or splitting nodes on the guide tree [15]. Given that we wanted to test for the status of the rodent malaria subspecies and that these have been previously defined on the basis of morphological characters and isozyme polymorphism [1], we considered all the subspecies as operational taxonomic units and constructed the guide tree with *BEAST (see methods for details of the *BEAST model; [11]). The topology of the guide tree generated by *BEAST is that in Figure 1, with *P. chabaudi* and *P. vinckei* forming a clade, *P. berghei* and *P. yoelii* forming another clade*,* and all the subspecies nesting within their corresponding species.

Using the guide tree obtained from *BEAST, we then performed the BPP analysis. To verify the stability of our results, we set up alternative models that tested for the effects of: (i) algorithm and fine-tuning parameters and (ii) population size (*θ*) prior (Additional files 2 and 3; see methods). In all cases, BPP overwhelmingly supported the fully-resolved guide tree (as in Figure 1) with posterior support very close to 1 (Additional files 2 and 3). Moreover, in all analyses, the secondary sampled models consistently collapsed nodes *e* or *h* in Figure 1 (similar results were obtained for a dataset with only 5 loci; data not shown; see methods and Additional files 2 and 3). Thus, our results were consistent across replicate runs and with different algorithms (and fine-tune parameters), indicating that mixing of the BPP rjMCMC algorithms was good.

#### Genealogical sorting index (gsi)

We used the genealogical sorting index [16] to validate the results from BPP. This method quantifies exclusive ancestry on a scale of 0 to 1, where 1 represents complete monophyly [12,16]. *gsi* can be calculated for single or multiple genes. As we wanted to test for the overall extent of exclusive ancestry for the rodent malaria subspecies, we computed *gsi* for multiple genes (*gsi*_*T*_) for each of the subspecies, using the genealogical sorting index web interface ( http://www.genealogicalsorting.org). The input for this software are gene trees, which we generated in BEAST as described in the methods. The great majority of *gsi*_*T*_ are above 0.7, (Additional file 4) with all but two (*P. y. killicki* and *P. y.* subsp.) being significant (see methods for significance test). This suggests a high level of exclusive ancestry for the majority of the subspecies (we obtained similar results for a 5 loci dataset; data not shown). Therefore, the results from BPP and *gsi* are robust and generally in agreement, suggesting that all 13 of the rodent malaria subspecies are independently evolving lineages.

### Divergence times and population sizes

As the fully resolved subspecies tree, preferred by BPP and *gsi*, reveals a high level of exclusive ancestry for the subspecies, we used the different subspecies as the operational taxonomic units for estimating a time-calibrated multi-locus species tree in *BEAST and obtained estimates for the effective population sizes through the same method.

A variety of approaches have been used to estimate divergence times in malaria parasites, and as yet there seems to be little consensus on the age of key divergences [13,19-21]. To reflect this uncertainty, we selected two calibrations as alternative priors for dates of common ancestry in rodent *Plasmodium*. The two calibrations we use are estimates for the root height of rodent malaria parasites and were published in [21]. In this paper, Pacheco *et al.* suggested a most recent common ancestor for the mitochondrial DNA of all rodent malaria at 8.32 million years ago (Mya; 95% Highest Posterior Density interval [HPD]: 5.10-12.60) and 14.2 Mya (9.96-18.89; see Table 3 in [21]). For simplicity, we denote the two calibrations as Pacheco2011-A and Pacheco2011-B, respectively. From these estimates, we derived gamma-distributed priors for the root of *coI* and *cytb* (see methods). Importantly, these estimates are consistent with the occurrence of malaria in lemurs in Madagascar, which could not have been acquired after the last terrestrial mammal colonization event (~20 Mya) [21-23] (see methods for a full description of the calibrations). We also used a third calibration using the *cytb* molecular clock inferred in [20], but estimates from this calibration fall within the Pacheco2011-A HPDs. Thus, we present results only for the Pacheco2011 calibrations.

**Table 3:** Divergence times as estimated by *BEAST

**node**	**Pacheco2011-A (Mya)**	**Pacheco2011-B (Mya)**	**Pacheco2011 combined 95% HPD (Mya)**
a*	7.3 (4.5; 11.1)	13.1 (9.0; 17.9)	4.5 – 17.9
b	2.3 (1.2; 3.6)	4.1 (2.5; 5.9)	1.2 – 5.9
c	0.6 (0.3; 0.9)	1.0 (0.5; 1.6)	0.3 – 1.6
d	0.4 (0.2; 0.7)	0.7 (0.4; 1.1)	0.2 – 1.1
e	0.1 (0.03; 0.2)	0.2 (0.07; 0.4)	0.03 – 0.4
f	5.1 (2.9; 8.0)	9.2 (5.7; 12.9)	2.9 – 12.9
g	0.6 (0.3; 0.9)	1.0 (0.6; 1.6)	0.3 – 1.6
h	0.4 (0.1; 0.6)	0.6 (0.3; 1.0)	0.1 – 1.0
i	3.8 (2.1; 5.9)	6.8 (4.3; 9.6)	2.1 – 9.6
j	2.6 (1.4; 4.0)	4.6 (2.8; 6.6)	1.4 – 6.6
k	2.2 (1.2; 3.4)	3.9 (2.5; 5.5)	1.2 – 5.5
l	0.4 (0.2; 0.8)	0.8 (0.5; 1.3)	0.2 – 1.3

*our posterior for the basal split of rodent *Plasmodium* (node *a*) differs slightly from our priors. This is because we set up calibrations on the root height of the linked mitochondrial gene tree, rather than on the root height of the species tree (i.e. including all loci).

The combined 95% HPD for the Pacheco2011 calibrations represent our best divergence time estimates. Nodes are labeled as in Figure 1. Values in brackets represent 95% HPD.

Table 3 shows the inferred divergence times depending on the different calibrations we used. As expected, the Pacheco2011-A resulted in younger estimates than Pacheco2011-B, with point estimates for the major splits between *P. berghei – P. yoelii* and between *P. chabaudi* – *P. vinckei* ranging from 1–6 and 3-13 Mya, respectively. From all analyses, it is clear that *P. chabaudi* and *P. vinckei* diverged much earlier than *P. berghei* and *P. yoelii*, with the latter divergence occurring simultaneously or after the divergence of the subspecies *P. v. brucechwatti*, *P. v. lentum* and *P. v. vinckei*.

We also used *BEAST to estimate the effective population sizes under the different calibrations. While the effective population sizes under the *BEAST model generally mixed well, the effective sample sizes (ESS) were often below 200 for the end population sizes (particularly for the Pacheco2011-B calibration), suggesting poor mixing of the MCMC algorithm. Thus, we treat these results with caution and discuss the population sizes only from a qualitative perspective for the tips of the tree (i.e. the subspecies) and not for the ancestral nodes. As expected, relative *BEAST estimates of effective population sizes generally reflect estimates of neutral genetic diversity (*π*_*S*_), and suggest that *P. berghei* and *P. v. vinckei* have the smallest population sizes, whereas *P. c. chabaudi*, *P. c. adami* and *P. y. yoelii* have the largest (Additional file 5).

### Molecular evolution

To better understand how selection is shaping the evolution of the genomes of rodent malaria parasites, we computed the following statistics for each gene (in DNAsp v5 [24]): McDonald-Kreitman (MK) tests (*α:* proportion of non-synonymous substitutions attributable to positive selection) [10]; Tajima’s *D* at synonymous sites [25], polymorphism (*π*_*A*_, *π*_*S*_ and *π*_*A*_*/π*_*S*_) and divergence (*K*_*A*_, *K*_*S*_ and *K*_*A*_*/K*_*S*_) [26]. To infer patterns of substitution independently for each lineage, we measured divergence from a putative ancestral sequence inferred by maximum likelihood for the *P. bergheiP. yoelii* and *P. chabaudi-P. vinckei* nodes, under a codon model using PAML v4.5 [27]. In this analysis we used either the species or the subspecies with the most genotypes (*P. c. chabaudi*, *P. v.* subsp. and *P. y. yoelii*) as the intraspecific groups. We also performed this analysis for *P. v. petteri* because this subspecies was isolated from the same location as *P. c. chabaudi* and *P. y. yoelii* and tested for significant differences between taxa in *π*_*A*_, *π*_*S*_, *π*_*A*_*/π*_*S*_, *K*_*A*_, *K*_*S*_ and *K*_*A*_*/K*_*S*_, using pair wise Wilcoxon rank sum tests (with *p-values* adjusted by Bonferroni correction).

**Figure 2:**
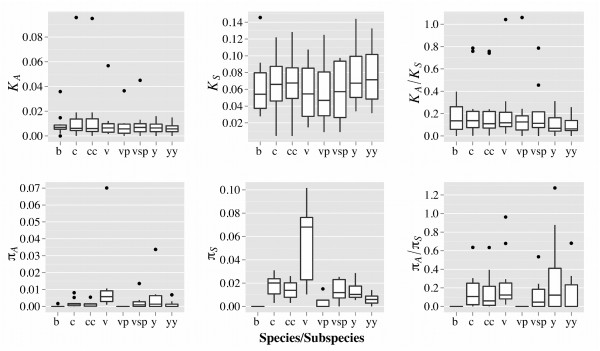
**Divergence and polymorphism in rodent malaria parasites.** Boxplots were constructed using values obtained for each of the 11 nuclear loci (Additional files 7, 10) and represent divergence (top row) and polymorphism (bottom) at non-synonymoys sites (left; *K*_*A*_*, π*_*A*_), synonymous sites (middle; *K*_*S*_*, π*_*S*_) and their ratio (right; *K*_*A*_/*K*_*S*_, *π*_*A*_/*π*_*S*_). Estimates were obtained for: *P. berghei* (b); *P. chabaudi* (c); *P. c. chabaudi* (cc); *P. vinckei* (v); *P. v. petteri* (vp); *P. v.* subsp. (vsp); *P. yoelii* (y); *P. y. yoelii* (yy).

For both *α* and Tajima’s D at silent sites, there was only one result significantly incompatible with a standard neutral model: *cyspro* in *P. chabaudi* (*p* = 0.016) and *hsp70* in *P. yoelii* (*p* < 0.05), respectively. The distribution of divergence (Figure 2) is similar across lineages with no significant differences between them. Although there were some neutrally evolving lineage-specific exceptions, *K*_*A*_*/K*_*S*_ is generally low (~0.2), suggesting that these loci are under purifying selection across most parasite lineages. Neutral polymorphism (*π*_*S*_) was generally between 0.2 and 3%, reaching 10% only if *π*_*S*_ was measured for *P. vinckei*. Additionally, polymorphism varies between lineages and significant differences could be detected, particularly for *P. vinckei* (Additional file 6). As other researchers have shown [14,28], polymorphism in *P. berghei* was extremely low and we only detected two non-synonymous SNPs in *cyspro* for the K173 and K173N genotypes (Additional file 7).

Finally, we used a maximum likelihood version of the MK test [29] to further test for variation in *α* between loci and/or lineages and to estimate mean *α* from multi-locus data. This software implements several likelihood models, which can be used to test different hypotheses about selection at loci of interest. We set up three models, in which: (i) *α* is constrained to zero at all loci, i.e. no adaptive evolution; (ii) *α* is a free-parameter common to all loci; and (iii) *α* can take a different value at each locus. We compared models using Akaike weights and estimated –α and 95% bootstrap intervals across loci as described in the methods. Compared to other loci, *cyspro* in *P. c. chabaudi* and *P. v.* subsp. shows extremely high divergence. This may reflect a substantial underlying change in its biology, and we therefore performed the analysis both including and excluding this locus.

We show that variation in *α* between loci is restricted to *P. c. chabaudi* and *P. v.* subsp. and is influenced by the presence of *cyspro* (Additional file 8). In the absence of *cyspro*, the best models always include *α* constrained to zero (model *i*) or as a free-parameter common to all loci (model *ii*), with model *i* having substantially higher Akaike weights for *P. v.* subsp. and *P. y. yoelii*, indicating there is little support for positive selection in these lineages. Additionally, the 95% bootstrap intervals for *α* were wide, and *α* did not vary significantly between lineages. Therefore, our results indicate that most loci either neutrally evolving or under purifying selection.

## Discussion

By sequencing multiple loci from 58 genotypes across four species of rodent *Plasmodium*, we clarify the evolutionary context for studying both the population genetics and the functional biology of this group of organisms. First, our results suggest that all subspecies are independently evolving lineages and that the number of rodent malaria species may be underestimated. Second, we suggest two different timelines for the evolution of rodent malaria parasites (with point estimates for the *P. berghei – P. yoelii* and the *P. chabaudi* – *P. vinckei* splits ranging from 1–6 and 3–13 Mya, respectively). Third, we find that most of the 11 nuclear loci used are under purifying selection, although there is some evidence that a potential vaccine candidate (*cyspro*) may evolve under positive selection. In agreement with other studies, we show that polymorphism is almost absent (but non-zero) in isolates of *P. berghei*.

All analyses suggest that the rodent malaria subspecies could be considered species (with the possible exception of the unnamed *P. y.* subsp.; Additional files 2, 3, and 4). However, this fine-scale division corresponds exactly to their geographic sampling ranges ( [1]; Table 1). Since the species delimitation approaches assume random mating within the delimited lineages and that this assumption is not met in the presence of population structure, we suggest that this conclusion be treated with caution [15]. Zhang *et al.*[17] recently used simulated data to test the effect of migration on the inferred species delimitation model, and showed that for sample sizes similar to ours, BPP will tend to infer a model with two species when migration between the populations is low (i.e. *M* < 1 migrant per generation) and a single species when migration is high (i.e. *M* > 10), with the posterior probability of inferring the two-species model decreasing sharply when 1 < *M <* 10. Thus, the migration parameter space for which we could have erroneously inferred multiple species appears to be rather reduced. Moreover, the extreme divergence between some subspecies, especially those within *P. vinckei* (*P. v. vinckei*, *P. v. brucechwatti* and *P. v. lentum*), clearly warrants careful attention, as these lineages are at least as divergent as *P. berghei* and *P. yoelii* (Table 3, Figure 1 and Additional file 9). Treating *P. vinckei* subspecies as species would also resolve the inconsistency between level of divergence and taxonomic rank of *P. v. vinckei* first detected in [14]. Regardless of taxonomy, these deep divergences need to be taken into careful consideration in experimental studies with *P. vinckei*. More broadly, the correct identification of independently evolving lineages (species) is of critical epidemiological relevance, as it can enable interbreeding populations to be identified, which is essential for the management of disease [30].

Perkins *et al.*[14] provided an earlier rodent malaria phylogeny, based on 7 loci and 19 genotypes. Our analyses, based on 11 loci and 58 genotypes, similarly confirms the original morphological and isozyme-based species classification [1]. The topology of our species tree broadly matches that of Perkins *et al.*[14], suggesting a robust rodent malaria phylogeny. We used two time-calibrations to estimate the divergence times of rodent *Plasmodium*. However, given we have little information about the divergence process of rodent malaria parasites or their hosts, we consider the full span of the combined 95% HPDs for both Pacheco2011 calibrations as the estimates which best reflect the available knowledge, i.e. the major splits in the rodent phylogeny occurred at roughly 4–18 (basal split; see [21]), 1–6 (*P. berghei – P. yoelii*) and 3–13 Mya (*P. chabaudi* – *P. vinckei*). These estimates coincide with a recent estimate for the divergence of the *Grammomys* genus (which includes known host species for rodent *Plasmodium*) [31].

Whilst polymorphism was generally low, *P. vinckei* displayed significantly more diversity than other taxa. However, this is because divergence between *P. vinckei*’s subspecies is much greater than for the *P. chabaudi* and *P. yoelii* subspecies. Accordingly, the level of polymorphism within *P. v.* subsp. (the *P. vinckei* subspecies with most available genotypes) is within the range of the other taxa. This suggests that, at least for *P. vinckei*, molecular evolution tools should be applied at the subspecies, rather than the species level, due to the deep divergences of its subspecies. Given this, care should be taken when generalizing observations from a single *P. vinckei* subspecies to the species level.

In contrast to *P. vinckei,* our analysis confirms previous reports that genetic diversity for *P. berghei* is very low, but not zero [14,28]. Based on the apparently low diversity in *P. berghei*, it has been suggested that the diversity detected in the past could have arisen in the laboratory (i.e. after isolation). We identified 2 polymorphisms across 6500 observed sites in *P. berghei*, which is much higher than the expected value (0.005 mutations) if these mutations had been acquired after isolation (assuming a star-shaped phylogeny for the genotypes, 60 years since parasite isolation and a mutation rate of 1.2x10^-8^ per site per year; [20]). This suggests that the observed mutations were probably present in the natural populations. Despite *P. berghei*’s key role as a model for the study of functional genomics [32], this near absence of polymorphism limits its utility for understanding the functional consequences of genetic variation. At the subspecies level, *P. c. chabaudi*, *P. y. yoelii* and *P. v.* subsp. show the highest diversity (in terms of *π*_*S*_), making these the best rodent models for the identification of genetic markers for quantitative-trait loci analysis. Whilst genotypes of *P. c. chabaudi* and *P. y. yoelii* have been used to identify drug resistance and virulence loci [33,34], *P. v.* subsp. has not been used in this type of study. However, given that average *π*_*S*_ for this species is twice that of *P. y. yoelii* and almost as high as for *P. c. chabaudi*, this subspecies may provide an alternative model for such studies.

The relative rates of protein evolution (*K*_*A*_*/K*_*S*_) did not differ between species/subspecies and suggest that almost all loci analyzed are evolving under some constraint, as would be expected for house-keeping loci. However, some loci do show very low levels of constraint in particular lineages (e.g. *rnabind* in *P. chabaudi*), possibly suggesting functional differences between lineages (Figure 2, Additional files 7 and 10). Accordingly, a comparison of bootstrap intervals failed to detect significant differences between lineages in maximum-likelihood estimates of *α*, and overall there is little evidence for positive selection. The lack of variation in mean *α* and *K*_*A*_*/K*_*S*_ between lineages is perhaps surprising given the apparently large differences seen in effective population size (both in the *BEAST analysis and in terms of *π*_*S*_) [35]. However, this may mostly reflect lack of power, as both the estimates of mean *α* and *K*_*A*_*/K*_*S*_ present large confidence intervals (Figure 2; Additional files 6, 7 and 10). Nevertheless, our mean estimates of *α* do increase with effective population size (as might be expected if positive selection is more effective at higher effective population sizes; Additional files 5 and 8) [35]. Additionally, using individual gene-wise MK analysis, we identified a single potential candidate for the action of strong positive selection: *cyspro* in *P. chabaudi*. As no correction for multiple tests was made, we treat this result with caution. Nevertheless, given that the *P. falciparum* ortholog of *cyspro* (falcipain-1) has been proposed as a possible vaccine candidate [36], if the functional role and selective patterns of *cyspro* are similar in *P. chabaudi* and *P. falciparum*, *P. chabaudi* could be a useful model to test the efficacy of vaccines targeting this locus and understand potential evolutionary responses by the parasite.

## Conclusions

The work here presented improves the utility of rodent *Plasmodium* as models for the study of malaria as it provides a much-needed evolutionary context for understanding biological aspects as the cross-reactivity of immune responses or parasite mating patterns. Moreover, while evolutionary genetics tools have rarely been used to study rodent *Plasmodium*, this approach has great utility for understanding the selective pressures shaping the evolution of malaria parasites and for generating hypotheses about the molecular interactions between parasites, hosts, and vectors.

## Methods

### Parasites and hosts

We used parasite genotypes from stabilates frozen as close as possible to the date of collection/arrival in Edinburgh (dates in Additional file 1) to avoid genotypes previously subjected to artificial selective pressures (intentionally or unintentionally) and minimize host passages. Cryopreserved parasite stabilates (−80°C) were defrosted and injected into MF1 male mice (in house supplier, University of Edinburgh) in 100 μl carrier solution [37]. When sufficient parasitaemia was visible in giemsa stained thin blood smears, blood samples were taken and DNA extracted (following [37]) for PCR and sequencing.

### PCR and sequencing

To sequence the 11 nuclear loci, we performed PCR reactions using primers designed for conserved regions (Additional file 11) of *P. berghei*, *P. chabaudi* and *P. yoelii* (sequences obtained from http://plasmodb.org). We treated PCR products with Exonuclease 1 (New England Biolabs, UK) and Shrimp Alkaline Phosphatase (Sigma-Aldrich, UK) to remove unused PCR primers and dNTPs, and then sequenced in both directions using BigDye reagents (Applied Biosystems, UK) on an ABI 3730 capillary sequencer (Gene Pool Sequencing Facility, Edinburgh). For most genes, more than 85% of genotypes provided sequence data (Table 2).

### Sequence analysis

We assembled sequences with Seqman (DNASTAR), inspected all polymorphisms manually and aligned sequences with ClustalW (in BioEdit [38]), with adjustments by eye. *Gdpgmp* was divided into intronic and exonic regions before analysis. We used GARD [39,40] in Datamonkey [41,42] to test for recombination at each locus, but there was no statistical support for recombination in any loci. We then generated preliminary gene trees with BEAST (v1.6.2, [43]), which placed two genotypes outside of the expected taxon: (i) *P. v. vinckei* v-52 groups with *P. c. chabaudi*; and (ii) *P. v. vinckei* v-67 groups with *P. y. nigeriensis*. This was consistent across all 11 genes, indicating past misidentification and/or labeling error, and these sequences were retained under the new species identification suggested by gene trees (see Additional file 12 for a representative gene tree). Interestingly, previous researchers had labeled *P. v. vinckei* v-67 as ‘*P. berghei*-like’ ( [44]; on the basis of morphology), which is similar to *P. y. nigeriensis*.

### Species delimitation

#### BPP models

BPP uses gamma priors G(*α,β*) on the population size parameters (*θ*) and on the age of the root in the species tree (*τ*_*0*_), with prior mean *α*/*β* and prior variance *α*/*β*^*2*^. The other divergence time parameters were assigned the Dirichlet prior (equation 2 in [15]). We performed two sets of analyses with BPP. First, we fixed the *θ* and *τ*_*0*_ priors (*θ ~* G(1.5,300) and *τ*_*0*_ ~ G(1.5,30)) and set up BPP runs using two algorithms that used different combinations of fine-tune parameters (Additional file 2). Second, using algorithm 0 (fine-tune ε = 5), we allowed the *θ* prior to vary *θ ~* G(1.5, 150) or *θ ~* G(1.5, 1500); Additional file 3). The *τ*_*0*_ prior was G(1.5,30) for all analyses and was selected based on estimates from [13,20]. We varied only the *θ* prior because, contrary to the *τ*_*0*_ prior, this has been shown to have a strong impact upon the speciation probabilities [17,18]. We ran each analysis in duplicate, for 5x10^6^ steps (sampling every 50), with a burn-in of 5x10^4^ and confirmed that the starting speciation model was different for replicate runs (this is important to test for the stability of the results).

#### gsi significance tests

The significance of *gsi*_*T*_ was evaluated by comparing the values obtained with the null hypothesis that the amount of exclusive ancestry observed is the same as might have been observed at random. This was done by generating 10000 permutations on the subspecies labels, while holding the tree constant, and computing *gsi*_*T*_ for each permutation. A *p-value* was then computed as the probability of randomly obtaining *gsi*_*T*_ values that are equal to or greater than the observed *gsi*_*T*_ value [16]. As our dataset is unbalanced and it is known that uneven sample sizes can lead to an underestimation of the *p-values* for small group sizes, we assessed significance at 0.001 [12,16,45,46].

We repeated both BPP and *gsi* analysis with a reduced dataset with 5 loci (*26 s*, *atpase*, *dhfr*, *exonuc*, *rnabind*; data not shown), to test if our results were robust to changes in number of loci used.

### *BEAST models

*BEAST (from the BEAST software package [11,43]) is an MCMC method that estimates the species tree directly from multi-locus sequence data, under the multispecies coalescent model. It assumes that discrepancies between gene trees are due to incomplete lineage sorting rather than gene flow and incorporates uncertainty in nucleotide substitution model parameters and the coalescent process.

We performed two independent replicate runs for each of the different analysis (i.e. guide tree and different calibrations), for 1.1x10^9^ states (sampling every 10^5^ states and excluding the first 10% as burn-in) using the entire nuclear dataset and the mitochondrial loci. We used the HKY [47] substitution model with the dataset partitioned into 3 codon positions, no site heterogeneity, substitution models for all nuclear loci linked (apart from the *gdpgmp* intron) and a strict clock. We applied a Yule process speciation prior for species branching rates and a piecewise linear and constant root model for population sizes. We assessed convergence by evaluating the sampled values for each parameter across time in Tracer v1.5 ( http://beast.bio.ed.ac.uk/Tracer). Except where indicated, ESS values were above 200, replicate runs converged and we combined tree and log files with LogCombiner v1.6.2 ( http://beast.bio.ed.ac.uk). We also performed a more complex analysis in which the best substitution model for each locus was inferred with jmodeltest (phylemon 2.0 webserver: http://phylemon.bioinfo.cipf.es/, [48-50]) and the molecular clock was set to strict or relaxed depending on the distribution of the coefficient of variation and the ucld.mean in a BEAST model with a relaxed clock. However, given that the results of this analysis are similar to those obtained using HKY and a strict clock for all loci, we present only the results for the later and simpler model.

#### Time calibrations

The Pacheco2011 calibrations are estimates for the root height of rodent malaria parasites obtained from [21]. In this paper, the authors calibrated a tree of malaria parasites from mammals, birds and reptiles at the *Papio*/*Macaca* divergence (i.e. assuming host-parasite co-divergence; Pacheco2011-A) and the latter plus the Human/*Macaca* divergence (Pacheco2011-B). Using the estimates from [21], we calibrated the root of *coI* and *cytb* for the rodent malaria parasites, using a gamma distributed prior, with the following parameterization: Pacheco2011-A – shape = 3.216578, scale = 1.258366 and offset = 4.272368; Pacheco2001-B – shape = 7.607868, scale = 0.9322701 and offset = 7.1074117.

### Maximum likelihood MK test

Due to small sample sizes, we compared models using AICc and calculated Akaike weights following [51] (Akaike weights represent the probability that a model is true, given that the true model is amongst those tested). To estimate –α and 95% confidence intervals across loci, we performed 1000 bootstraps on the results from model *ii*. We used the resulting distributions to test whether –α significantly differed between lineages (i.e. to detect lineage-specific selection patterns). We performed this analysis for the subspecies *P. c. chabaudi*, *P. v.* subsp. and *P. y. yoelii*, using polymorphism and divergence counts obtained as described under ‘molecular evolution’ in the results (see Additional file 10).

## Animal ethics statement

All the protocols involving mice passed an ethical review process and were approved by the U.K. Home Office (Project License 60/4121). Work was carried according to the Animals (Scientific Procedures) Act, 1986.

## Competing interests

The authors declare that they have no competing interests.

## Author contributions

RSR, SER and DJO designed the study, RSR collected the DNA sequence data, RSR and DJO performed the analysis. All authors contributed towards writing the manuscript and approved its final version.

## Supplementary Material

Additional file 1 **Summary of the rodent malaria parasite genotypes used.** Lines in bold represent clones. Location: CAM - Cameroon; CAR - Central African Republic; CON - Congo; DRC - Democratic Republic of the Congo; NIG - Nigeria. Mixed infection: nk - not known; Pcc - *P. c. chabaudi*; Pv - *P. vinckei*; Pvp - *P. v. petteri*; Pyk - *P. y. killicki*; Pysp - *P. y.* subsp.; Pyy - *P. y. yoelii*. ? - information is uncertain. Note that where isolate and arrival dates are equal is because infected *T. rutilans* were shipped to Edinburgh. Click here for file

Additional file 2 **Posterior probabilities for the speciation models sampled by BPP, using the different algorithms and fine-tune parameters (ε,*a *and *m*).** Priors were kept constant: *θ~*G(1.5,300) and *τ*_*0*_~G(1.5,30). Each speciation model is represented using 0–1 flags for the interior nodes, with 0 indicating a collapsed node and 1 a resolved node. Nodes are ordered as in Figure 1, with the first flag corresponding to node *a*, the second to node *b*, and so on up to node *l*. Each cell has the posterior probability for two replicate runs of BPP, which started with different speciation models. NS: model not sampled. Click here for file

Additional file 3 **Posterior probabilities for the speciation models sampled by BPP, using different values for the*θ * prior.** We used algorithm 0 with fine-tune ε = 5 for this analysis. Speciation model and cell content are as described for Additional file 2. Click here for file

Additional file 4 **Ensemble genealogical sorting index (*gsiT*) for the independently evolving lineages identified by BPP.** Click here for file

Additional file 5 Population sizes for the rodent malaria subspecies as inferred by *BEAST. Point estimates (and 95% HPDs) are shown for each calibration. Pb - *P. berghei*; Pca - *P. c. adami*; Pcc - *P. c. chabaudi*; Pcsp - *P. c.* subsp.; Pvb - *P. v. brucechwatti*; Pvl - *P. v. lentum*; Pvp - *P. v. petteri*; Pvsp - *P. v.* subsp.; Pvv - *P. v. vinckei*; Pyk - *P. y. killicki*; Pyn - *P. y. nigeriensis*; Pysp - *P. y.* subsp.; Pyy - *P. y. yoelii.* Click here for file

Additional file 6 **Pairwise comparisons of*π***_***A***_**,*π***_***S***_**and*π***_***A***_***/π***_***S***_**across taxa.***p-values* for pairwise Wilcoxon tests with Bonferroni corrections are shown. *p* < 0.05 are highlighted in bold. b - *P. berghei*, c - *P. chabaudi*, cc - *P. c. chabaudi*, v - *P. vinckei*, vp - *P. v. petteri*, y - *P. yoelii*, yy - *P. y. yoelii*. Click here for file

Additional file 7 **Summary of the analysis of polymorphism and divergence in the four species of rodent malaria across the 11 loci.** n - number of genotypes analysed; MK - McDonald-Kreitman test; * represents statistically significant MK tests or Tajima's D; estimates in bold are outliers in Figure 2. Variation in the number of sequenced genotypes (Table 2) and an absence of polymorphism and/or divergence in some taxa meant we could not obtain these statistics for every gene in all lineages. Click here for file

Additional file 8 **Best likelihood models and estimates of the proportion of adaptive substitutions across loci (α¯).** Divergence was measured to the inferred ancestral sequence of the *P. chabaudi-P. vinckei* clade (*P. c. chabaudi* and *P. v.* subsp.) or the *P. berghei-P. yoelii* clade (*P. y. yoelii*). The best (highest Akaike weight) and secondary models are shown, with Akaike weights in brackets. Estimates of α¯ (and 95% CI) across loci were obtained from 1000 bootstraps on the results of model ii. Likelihood models: i) *α* is constrained to zero at all loci, i.e. no adaptive evolution; ii) *α* is a free-parameter common to all loci; and iii) *α* can take a different value at each locus. Click here for file

Additional file 9 Species tree inferred by *BEAST under the Pacheco2011-A calibration. Node labels are posterior probabilities and node bars represent 95% Highest Posterior Densities on the height of each node. Axis is in million years ago (Mya). Click here for file

Additional file 10 **Summary of the analysis of polymorphism and divergence for the subspecies of rodent malaria across the 11 loci.** Legend as for Additional file 7. Click here for file

Additional file 11 **Primer sequences (5’-3’) used in PCR for the selected loci.** PCR cycling conditions: 95°C for 3 min.; 10 cycles of: 94°C for 30s, 57°C for 30s (−1°C per cycle), 72°C for 1.5 min.; 35 cycles of: 94°C for 30s, 47°C for 30s, 72°C for 1.5 min.; 72°C for 3 min. Click here for file

Additional file 12 **Gene tree inferred by BEAST for*dhfr*.** Node labels are posterior probabilities. We chose to present the *dhfr* gene tree, as this is one of the locus for which we obtained sequence data for more isolates (56, as for *rnabind*) and for which node support was stronger. This is an example of the gene trees we obtained and we note that the branch lengths and their order may not be the same as for the multi-locus analysis or other gene trees. Therefore, the gene tree does not necessarily reflect relatedness between genotypes within each subspecies. Click here for file
