# Supplementary material for: Molecular evolution and phylogenetics of rodent malaria parasites
Source: BMC Evol Biol. 2012 Nov 14;12:219. doi: 10.1186/1471-2148-12-219 (PMC3538709; doi:10.1186/1471-2148-12-219)
Supplement: Additional file 2 — Posterior probabilities for the speciation models sampled by BPP, using the different algorithms and fine-tune parameters (ε,a and m). Priors were kept constant: θ~G(1.5,300) and τ0~G(1.5,30). Each speciation model is represented using 0–1 flags for the interior nodes, with 0 indicating a collapsed node and 1 a resolved node. Nodes are ordered as in Figure 1, with the first flag corresponding to node a, the second to node b, and so on up to node l. Each cell has the posterior probability for two replicate runs of BPP, which started with different speciation models. NS: model not sampled. [file 1471-2148-12-219-S2.pdf]

Additional File 3. Posterior probabilities for the speciation models sampled by BPP, using the different algorithms and fine-tune parameters (ε, *a* and *m*). Priors were kept constant: θ~G(1.5,300) and τ_0~G(1.5,30). Each speciation model is represented using 0-1 flags for the interior nodes, with 0 indicating a collapsed node and 1 a resolved node. Nodes are ordered as in Figure 1, with the first flag corresponding to node *a*, the second to node *b*, and so on up to node *l*. Each cell has the posterior probability for two replicate runs of BPP, which started with different speciation models. NS: model not sampled.

**speciation model**	**algorithm 0**		**algorithm 1**			
	**ε = 5**	**ε = 20**	***a* = 0.5; *m* = 1**	***a* = 0.5; *m* = 2**	***a* = 2; *m* = 1**	***a* = 2; *m* = 2**
111111111111	0.997; 0.996	1; 1	0.996; 0.991	0.998; 1	0.995; 0.999	0.994; 0.986
111101111111	2.7E-3; 3.7E-3	NS	4.1E-3; 8.7E-3	2.1E-3; NS	5.5E-3; 1.2E-3	6.2E-3; 0.014
