# Supplementary material for: Molecular evolution and phylogenetics of rodent malaria parasites
Source: BMC Evol Biol. 2012 Nov 14;12:219. doi: 10.1186/1471-2148-12-219 (PMC3538709; doi:10.1186/1471-2148-12-219)
Supplement: Additional file 3 — Posterior probabilities for the speciation models sampled by BPP, using different values for theθ prior. We used algorithm 0 with fine-tune ε = 5 for this analysis. Speciation model and cell content are as described for Additional file 2. [file 1471-2148-12-219-S3.pdf]

Additional File 4. Posterior probabilities for the speciation models sampled by BPP, using different values for the *θ* prior. We varied only the θ prior because, contrary to the τ_0 prior, this has been shown to have a strong impact upon the speciation probabilities (references 18 and 19 in main text). We used algorithm 0 with fine-tune $\varepsilon = 5$ for this analysis. Speciation model and cell content are as described for Table S3.

**speciation model**	**G(1.5, 1500)**	**G(1.5, 150)**
111111111111	0.939; 0.939	0.988; 0.975
111101111111	0.010; 0.005	0.012; 0.025
111111101111	0.050; 0.061	NS
