# Supplementary material for: Molecular evolution and phylogenetics of rodent malaria parasites
Source: BMC Evol Biol. 2012 Nov 14;12:219. doi: 10.1186/1471-2148-12-219 (PMC3538709; doi:10.1186/1471-2148-12-219)
Supplement: Additional file 4 — Ensemble genealogical sorting index (gsiT) for the independently evolving lineages identified by BPP. [file 1471-2148-12-219-S4.pdf]

Additional File 5. Ensemble genealogical sorting index (*gsiT*) for the independently evolving lineages identified by BPP.

**species**	***gsiT*** **(*p-values*)**
P. berghei	0.8333 (<0.001)
P. c. adami	0.4433 (<0.001)
P. c. chabaudi	0.8464 (<0.001)
P. c. subsp.	0.4163 (<0.001)
P. v. brucechwatti	0.9167 (<0.001)
P. v. lentum	0.9167 (<0.001)
P. v. petteri	0.7407 (<0.001)
P. v. subsp.	0.7855 (<0.001)
P. v. vinckei	0.9167 (<0.001)
P. y. killicki	0.5986 (0.0954)
P. y. nigeriensis	0.6667 (<0.001)
P. y. subsp.	0.3308 (0.0854)
P. y. yoelii	0.7613 (<0.001)
