# Supplementary material for: Molecular evolution and phylogenetics of rodent malaria parasites
Source: BMC Evol Biol. 2012 Nov 14;12:219. doi: 10.1186/1471-2148-12-219 (PMC3538709; doi:10.1186/1471-2148-12-219)
Supplement: Additional file 8 — Best likelihood models and estimates of the proportion of adaptive substitutions across loci (α¯). Divergence was measured to the inferred ancestral sequence of the P. chabaudi-P. vinckei clade (P. c. chabaudi and P. v. subsp.) or the P. berghei-P. yoelii clade (P. y. yoelii). The best (highest Akaike weight) and secondary models are shown, with Akaike weights in brackets. Estimates of α¯ (and 95% CI) across loci were obtained from 1000 bootstraps on the results of model ii. Likelihood models: i) α is constrained to zero at all loci, i.e. no adaptive evolution; ii) α is a free-parameter common to all loci; and iii) α can take a different value at each locus. [file 1471-2148-12-219-S8.pdf]

Additional File 10. Best likelihood models and estimates of the proportion of adaptive substitutions across loci ($\overline{\alpha}$). Divergence was measured to the inferred ancestral sequence of the *P. chabaudi-P. vinckei* clade (*P. c. chabaudi* and *P. v.* subsp.) or the *P. berghei-P. yoelii* clade (*P. y. yoelii*). The best (highest Akaike weight) and secondary models are shown, with Akaike weights in brackets. Estimates of $\overline{\alpha}$ (and 95% CI) across loci were obtained from 1000 bootstraps on the results of model ii. Likelihood models: i) α is constrained to zero at all loci, i.e. no adaptive evolution; ii) α is a free-parameter common to all loci; and iii) α can take a different value at each locus.

taxa	dataset	best model	secondary model	$\overline{\alpha}$
P. c. chabaudi	all loci	iii (1.00)	-	0.640 (0.212; 0.874)
	w/out *cyspro*	ii (0.65)	i (0.33)	0.456 (0.061; 0.769)
P. vinckei subsp.	all loci	iii (0.97)	i (0.03)	0.165 (-0.120; 0.516)
	w/out *cyspro*	i (0.73)	ii (0.27)	0.279 (-0.067; 0.698)
P. y. yoelii	all loci	i (0.74)	ii (0.26)	-0.261 (-1.189; 0.516)
	w/out *cyspro*	i (0.70)	ii (0.30)	-0.524 (-1.939; 0.470)
