# Supplementary material for: Molecular evolution and phylogenetics of rodent malaria parasites
Source: BMC Evol Biol. 2012 Nov 14;12:219. doi: 10.1186/1471-2148-12-219 (PMC3538709; doi:10.1186/1471-2148-12-219)
Supplement: Additional file 9 — Species tree inferred by *BEAST under the Pacheco2011-A calibration. Node labels are posterior probabilities and node bars represent 95% Highest Posterior Densities on the height of each node. Axis is in million years ago (Mya). [file 1471-2148-12-219-S9.pdf]

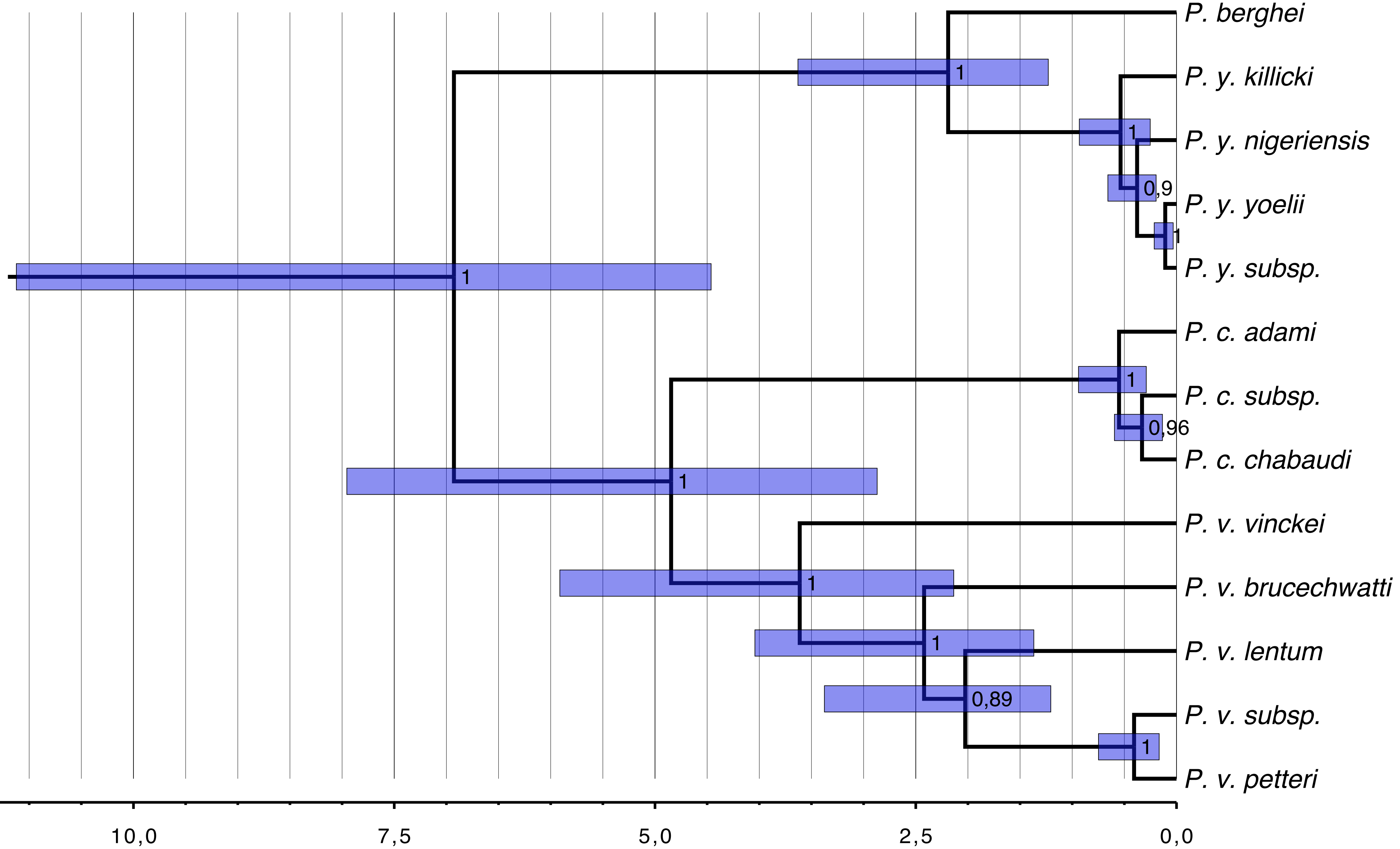

Additional File 11. Species tree inferred by *BEAST under the Pacheco2011-A calibration. Node labels are posterior probabilities and node bars represent 95% Highest Posterior Densities on the height of each node. Axis is in million years ago (Mya).
