# Supplementary material for: Molecular evolution and phylogenetics of rodent malaria parasites
Source: BMC Evol Biol. 2012 Nov 14;12:219. doi: 10.1186/1471-2148-12-219 (PMC3538709; doi:10.1186/1471-2148-12-219)
Supplement: Additional file 11 — Primer sequences (5’-3’) used in PCR for the selected loci. PCR cycling conditions: 95°C for 3 min.; 10 cycles of: 94°C for 30s, 57°C for 30s (−1°C per cycle), 72°C for 1.5 min.; 35 cycles of: 94°C for 30s, 47°C for 30s, 72°C for 1.5 min.; 72°C for 3 min. [file 1471-2148-12-219-S11.pdf]

Additional File 2. Primer sequences (5'-3') used in PCR for the selected loci. PCR cycling conditions: 95ºC for 3 min.; 10 cycles of: 94ºC for 30s, 57ºC for 30s (-1ºC per cycle), 72ºC for 1.5 min.; 35 cycles of: 94ºC for 30s, 47ºC for 30s, 72ºC for 1.5 min.; 72ºC for 3 min.

**gene**	**forward primer**	**reverse primer**
26s	ATCATCAGGGGAACATTTACCATCGA	ATGCCATTCCTTCAACTGTGGCAAT
atpase	ATTGATACTCCTGGACAAATAGAA	TGACATCTTTGTCTTCTCCATATTG
cons	TGATCACAGTTTAAGTGTATATGACAG	TTTCTCTGCCTTTATCATGGTAC
cyspro	CAATGAAATGGTAGGTAAAAATGGT	CCATGAATTTCTAATGATCCAGTAAT
dhfr	CAAGATGATAGAACAGGTGTTGGTG	CCCAATACATGTATAAATTCAGCTG
exonuc	GTAGTGCGTCCTATGGATATTTA	GAATTAGCTCTTTTGATATCGGTCT
gdpgmp	TGAAGGGATAAAACATTTATGGCCT	GTGTATTGCTTGTCCTATGGAAACG
glurna	AAGTTGTTACTCGATTTCCACC	GGCATGCAAAATCATAAGTTGGAT
hsp70	TTCCTATGATTATGTTGTGGATCA	CGTGTAACTGTTTCTTGTAAGC
metrans	TATTACCAGCAAGTTTTAGAGC	GGAATTGTACCAGTAAATCCTT
rnabind	GGAAAGTATGTCTAGGTATTCCAAC	GGTCTGCTATCTCCTAATTGTGA
