# Supplementary material for: Molecular evolution and phylogenetics of rodent malaria parasites
Source: BMC Evol Biol. 2012 Nov 14;12:219. doi: 10.1186/1471-2148-12-219 (PMC3538709; doi:10.1186/1471-2148-12-219)
Supplement: Additional file 12 — Gene tree inferred by BEAST fordhfr. Node labels are posterior probabilities. We chose to present the dhfr gene tree, as this is one of the locus for which we obtained sequence data for more isolates (56, as for rnabind) and for which node support was stronger. This is an example of the gene trees we obtained and we note that the branch lengths and their order may not be the same as for the multi-locus analysis or other gene trees. Therefore, the gene tree does not necessarily reflect relatedness between genotypes within each subspecies. [file 1471-2148-12-219-S12.pdf]

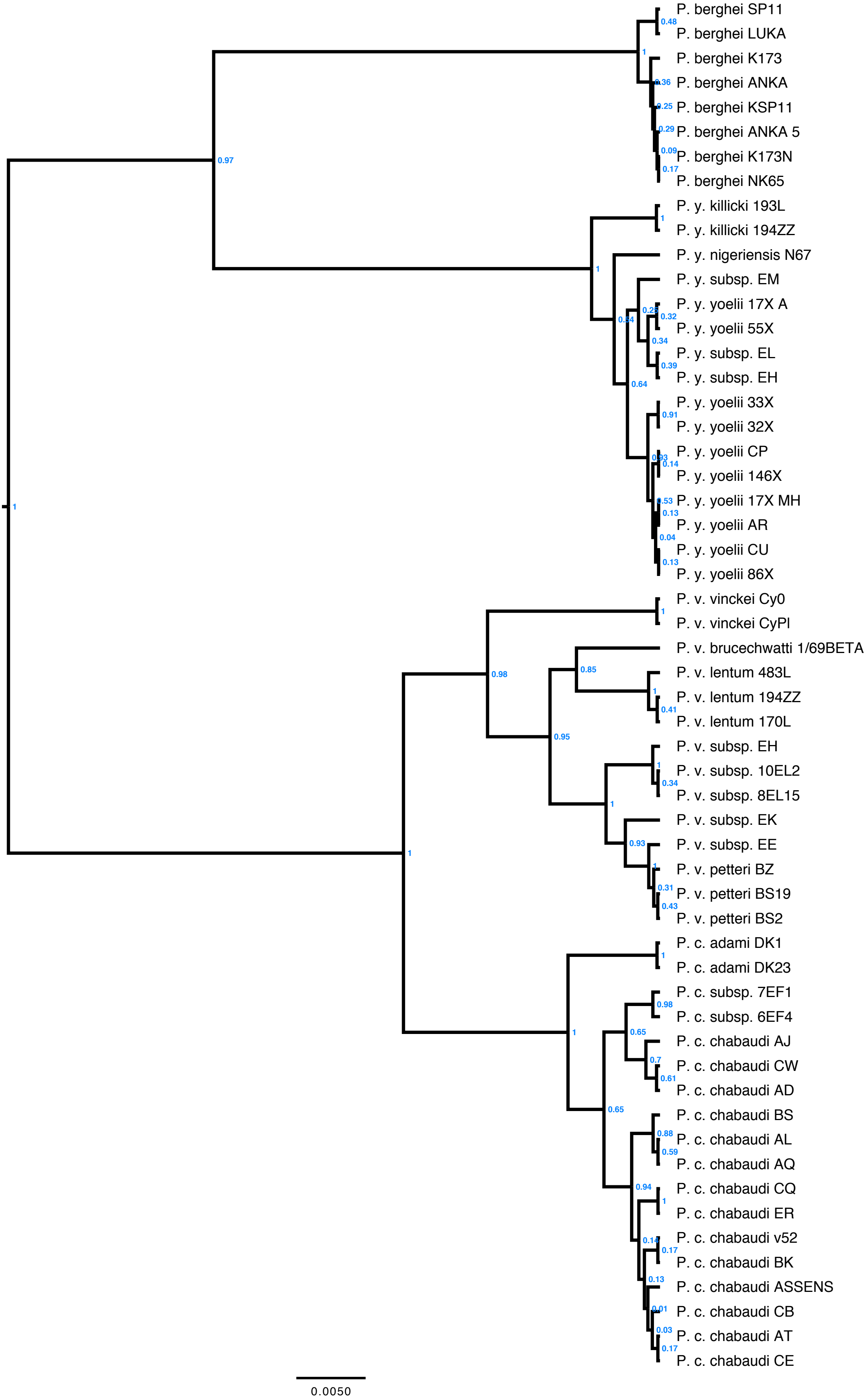

Additional File 12. Gene tree inferred by BEAST for *dhfr*. Node labels are posterior probabilities. We chose to present the *dhfr* gene tree, as this is one of the locus for which we obtained sequence data for more isolates (56, as for *rnabind*) and for which node support was stronger. This is an example of the gene trees we obtained and we note that the branch lengths and their order may not be the same as for the multi-locus analysis or other gene trees. Therefore, the gene tree does not necessarily reflect relatedness between genotypes within each subspecies.
